# Sending out an SOS - the bacterial DNA damage response

**DOI:** 10.1590/1678-4685-GMB-2022-0107

**Published:** 2022-10-10

**Authors:** Marco A. Lima-Noronha, Douglas L. H. Fonseca, Renatta S. Oliveira, Rúbia R. Freitas, Jung H. Park, Rodrigo S. Galhardo

**Affiliations:** 1 Universidade de São Paulo, Instituto de Ciências Biomédicas, Departamento de Microbiologia, São Paulo, SP, Brazil.

**Keywords:** DNA damage, SOS response, mutagenesis, TLS polymerases, mobile genetic elements

## Abstract

The term “SOS response” was first coined by Radman in 1974, in an intellectual effort to put together the data suggestive of a concerted gene expression program in cells undergoing DNA damage. A large amount of information about this cellular response has been collected over the following decades. In this review, we will focus on a few of the relevant aspects about the SOS response: its mechanism of control and the stressors which activate it, the diversity of regulated genes in different species, its role in mutagenesis and evolution including the development of antimicrobial resistance, and its relationship with mobile genetic elements.

The SOS response is a cellular mechanism induced by agents that threaten DNA integrity in prokaryotes that aids cell survival under stressful situations, since an unrepaired DNA damage may lead to deleterious mutations or even cell death. Cells are constantly exposed to environments that may contain DNA-damaging agents. These agents can be either a physical agent such as UV light and ionizing radiation, or a chemical compound such as alkylating and crosslinking agents. However, the threats that a cell has to face are not only external but also internal, such as the reactive oxygen species (ROS), metabolic byproducts that cause DNA damage.

Miroslav Radman used the distress signal “SOS” to define how bacterial cells sense genome instability, while studying DNA damage and replication blockages in *Escherichia coli* (Radman, 1974). This phenomenon triggers a pathway of physiological responses to deal with these adverse conditions, mainly DNA damage repair and/or tolerance and mutagenesis. Pathways induced by SOS include damage repair and tolerance mechanisms such as nucleotide excision repair (NER), photoreactivation, homologous recombination (HR) and translesion synthesis (TLS) (Erill et al., 2006). Despite the induction of pathways that promote DNA integrity in an error-free manner, there is also the involvement of error-prone elements in this response, responsible for improving cell survival under severe DNA damage, however exhibiting elevated mutagenesis as a consequence (Henrikus et al., 2018). SOS is subject to complex regulation controlled by the *lexA* and *recA* gene products, due to its mutagenic potential.

## Fundamentals of SOS response regulation

Induction of the SOS regulon is triggered by single-stranded DNA (ssDNA) present in the cell as a consequence of replication and repair of damaged DNA (Sassanfar and Roberts, 1990). Briefly, this response is regulated by the LexA and RecA proteins in which the former plays a role as a transcriptional repressor by binding to the promoter region of genes controlled by this regulon, and the latter functions as a positive regulator of the system (Little et al., 1980; Little, 1983, 1991; Aksenov, 1999).

## LexA - a self-cleaving repressor

Regulation of SOS response genes depends on transcriptional repression by the LexA protein, which binds to an operator sequence, within the promoter, known as the SOS box (Walker, 1984) and prevents RNA polymerase binding and transcription (Berg, 1988). LexA functions as a repressor in the form of a dimer consisting of two domains joined by a peptide linker: a DNA-binding domain located in the amino-terminal (NTD) and serine protease domain located in the carboxi-terminal (CTD). The CTD domain plays a role in the homodimerization of LexA (Zhang et al., 2010).

LexA repressor undergoes self-cleavage under SOS-inducing conditions (Slilaty et al., 1986). In *E. coli*, the enzyme has a conserved serine-lysine catalytic domain that self-cleaves its peptide bond between Ala84-Gly85 near the middle of the protein, thus losing its repressor function (Little, 1991). Structural studies in *E. coli* have shown that the CTD domain can be found in two different conformations: a basal cleavage-incompetent conformation and a cleavage-proficient conformation (Luo et al., 2001). *In vivo*, LexA self-cleavage occurs when it interacts with activated RecA protein (RecA*) (Little *et al.*, 1980).

## RecA - a DNA damage sensor

RecA protein is a key player in DNA repair, being required not only for SOS induction, but for homologous recombination and translesion synthesis as well. In the absence of ATP, RecA is found as monomers that are capable of associating with ssDNA, being able to protect DNA strand from degradation but staying in a functionally-inactive conformation (Yu and Egelman, 1992). When ATP molecules are available, the RecA-ssDNA complex is converted to the functionally-active conformation: RecA* nucleoprotein filament, a structure functioning as a co-protease responsible for inducing self-cleavage of LexA (Cox, 2007). This structure has many other functions, such as searching for homologous dsDNA to promote homologous recombination (Tsang et al., 1985). The RecA*-stimulated auto-cleavage of LexA expose previously inaccessible residues, facilitating proteolytic degradation of both fragments (Neher et al., 2003). Once LexA protein levels start to decrease, expression of SOS genes is triggered (Little and Mount, 1982).

## SOS response in a nutshell

**Figure 1 -:**
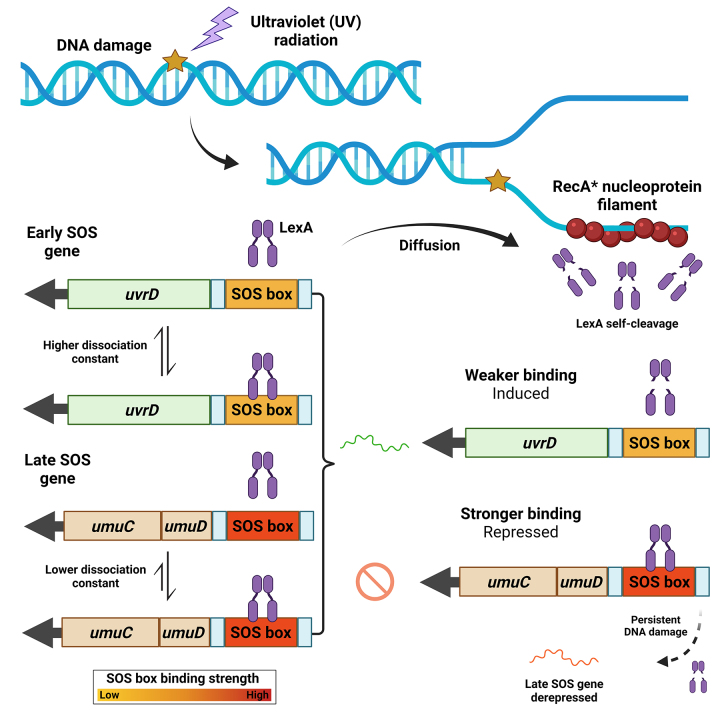
Model of SOS response activation. The presence of DNA damage may block DNA replication and expose ssDNA within the cell. RecA protein associates with ssDNA, assuming its functionally-active conformation: RecA* nucleoprotein filament. This protein complex is responsible for inducing LexA self-cleavage, thus enabling transcription of SOS regulated genes. LexA repressor displays a dynamic of binding/dissociating with its target sequence and can only be cleaved once it is dissociated from DNA. Note that a stronger SOS box implies a lower dissociation constant, meaning that in this scenario, LexA is more likely to be associated with DNA and thus repressing its target. Therefore, gene expression can be modulated by SOS box strength: the weaker the operator strength, the sooner a gene will be expressed.

Most of the findings regarding SOS response regulation and dynamics were made using the model bacterium *E. coli*. Once DNA breaks or other types of damage emerge within the cell, RecA monomers readily associate with ssDNA assuming its active form (RecA*), inducing LexA self-cleavage which causes it to dissociate from SOS-regulated promoters, thereby relieving repression of the SOS regulon (Figure 1). One important aspect of SOS dynamics is that *lexA* itself is an SOS gene, thus generating a negative-feedback loop to re-establish repression after the induction signal is ceased. Besides that, LexA is constantly expressed during late SOS to ensure that SOS induction is interrupted once stress signal decreases and LexA degradation is not favored anymore (Walker, 1984).

The dynamics of the SOS response can be manipulated by proteins that interact with RecA filament and modulate the time of induction and recovery rate of the response (Lusetti et al., 2004). The inhibitor of RecA is the RecX protein, which at low concentrations can suppress many RecA functions (Stohl et al., 2003) and blocks RecA filament polymerization (Drees et al., 2004), leading to filament dismantling (Ragone et al., 2008).

The response is orchestrated according to several variables, including the extent in which DNA was damaged and the time passed since such damage was identified (Courcelle et al., 2001; Quillardet et al., 2003), in such a way that SOS-regulated genes have different timing and levels of induction. Housekeeping and error-free repair processes comprise the initial phase of SOS, such as NER and homologous recombination. SulA protein, a division inhibitor, allows the bacterium to complete DNA repair before finalizing its cell division. Lastly, if the damage was severe and remains unrepaired, TLS-polymerases are induced leading to elevated mutagenesis but allowing replication to resolve, thus improving cell survival (Henrikus et al., 2018). It is also important to point out that RecA-mediated cleavage of LexA occurs when LexA is DNA-free but not when bound to its target DNA (Butala et al., 2011; Kovačič et al., 2013), adding more complexity to the timing of expression of SOS-regulated genes.

The dynamics of SOS genes in *E. coli* is also influenced by the strength of different SOS boxes (Figure 1). Usually, the consensus SOS box sequence displays a higher affinity for LexA binding since its sequence is a palindrome and optimal for LexA association: TACTG(TA)_5_CAGTA. Any modification within this sequence may interfere with LexA affinity for a given operator. The heterology index (HI) measures how much an SOS box differs from the consensus: the higher the HI value, the lower LexA affinity for the operator, as shown in *E. coli* (Lewis et al., 1994; Fernández de Henestrosa et al., 2000) and in *Salmonella enterica* (Mérida-Floriano et al., 2021). This process corroborates the idea that affinity of LexA is also an important factor, since lower affinity implies an earlier transcriptional derepression, consequently regulating genes that should be expressed early or late in the SOS response. 

## DNA damage response heterogeneity

To this date, the vast majority of studies measuring SOS induction have been using the “uniform expression model” (McCool et al., 2004). In this model, it is not clear whether the activity of a particular promoter is equally distributed across cells in a population or has a different expression for a subpopulation of cells (Kenyon and Walker, 1980; Salles and Defais, 1984). Measuring the activity of SOS regulated promoters in transcriptional fusions to reporter genes is an example that represents a population average and relies on the uniform expression model.

However, fluorescence microscopy studies to assess SOS induction at the single cell level pointed to limitations of measuring SOS induction at the population level (McCool et al., 2004; Britton et al., 2007; Jones and Uphoff, 2021). In this methodology, fluorescent proteins are fused to an SOS regulated gene and through microscopy analysis, rather than a population measurement, it is possible to determine subpopulations of cells displaying a variety of SOS induction patterns. This model is called the “two population model” and has shown how heterogeneous the induction of the SOS response within a cell population may be, allowing a much more accurate and comprehensive analysis for quantifying the SOS response.

## DNA damages and other stressors leading to SOS induction

An SOS response is triggered when single-stranded DNA (ssDNA) is present in the cell, which is one of the consequences of DNA damage. Repair of double-stranded DNA (dsDNA) breaks is a fundamental aspect of genome conservation. These potentially lethal lesions frequently occur during DNA replication (Pennington and Rosenberg, 2007). The enzymes RecA and RecBCD are the initiators required for double-strand breaks (DSBs) repair and homologous recombination. The type of DNA damage determines in which state the SOS response is triggered. These two enzymes at the same time degrade and unwind DNA from DSB *in vitro* (Chaudhury and Smith, 1985; Anderson and Kowalczykowski, 1997).

Several stresses, including quinolone treatment, high pressure and radiation lead to SOS induction as a result of DSB (Anderson and Kowalczykowski, 1997; Anderson and Kowalczykowski, 1998). RecBCD, responsible for processing DSBs, is a molecular machinery that binds to the damaged site and initiates the unwinding of the double-helix (Singleton et al., 2004). RecB is a helicase coupled to an endonuclease domain that initially degrades the 3’-tail more efficiently than the 5’-tail. RecC splits the DNA strands to each helicase (RecB and RecD) and scans for a recombinational hotspot, known as Chi (ꭓ) site (5’ GCTGGTGG 3’), where it can bind to and prevent further degradation from the 3’ end strand (Anderson and Kowalczykowski, 1997,^1998^). 

This event results in an ssDNA loop in the 3’ end strand to which RecA can be loaded. At the same time, RecD helicase is able to access the nuclease site more frequently, leading to a higher degradation rate of the 5’ end strand (Singleton et al., 2004). The classical DSB repair mechanism in *E. coli* occurs through homologous recombination (HR), which is dependent on homologous fragments from either exogenous DNA or a recently duplicated sequence after DNA replication. The nucleoprotein filament (ssDNA-RecA) from the 3’ end strand interacts with homology sequences, forming a heteroduplex structure with the intact dsDNA. Finally, DNA polymerase uses the complementary strand as a template to reconstitute double-stranded DNA and repair the DSB damage (Danilowicz et al., 2021).

DNA lesions can produce chemical alteration of the base structure, modifying the coding sequence of the molecule. The best example of base damaging agent is ultraviolet radiation, which leads to photochemical reactions between neighboring bases (Sassanfar and Roberts, 1990). It was found that in *E. coli* the *recF* pathway proteins, such as RecF, RecO, and RecR, are necessary to restore replication after UV radiation-induced damage. *recF*, *recO*, and *recR* mutants have enhanced sensitivity to DNA damage and show delayed SOS induction. RecFOR complex proteins stabilize and strengthen the binding of RecA (Bork et al., 2001; Rangarajan et al., 2002).

Replication forks frequently stall due to physical blockages. RecA* activation following replication blockage requires RecFOR complex for processing. There are several pathways supported by genetic evidence for homologous recombination and post replication repair in *E. coli* and the fact that the *recA* gene is required in all of these pathways suggests that other genes involved in the process of repair and recombination provide activities that help RecA. It is essential for genomic integrity that accurate replication recovery occurs after DNA damage and repair (Tseng Y-C *et al.*, 1994). 

Antibiotics that do not interfere directly with DNA replication may also induce the SOS response. Penicillin and related beta-lactams interfere with peptidoglycan metabolism by disturbing the activity of penicillin-binding proteins (PBPs). Impairment of PBPs activity by beta-lactams causes the induction of the two-component signal transduction system DpiBA in *E. coli*. DpiA has affinity to AT rich sequences and interferes with DnaA and DnaB binding at the replication origin, leading to SOS activation (Miller et al., 2003, 2004; Cho et al., 2014). 

In pathogens such as *Staphylococcus aureus* and *Pseudomonas aeruginosa,* the SOS response is involved in the mutagenesis leading to antibiotic resistance (Blázquez et al., 2006; Cirz et al., 2006, 2007; Maiques et al., 2006). SOS induction by antibiotics has many important implications, since it can increase error-prone polymerases that mediate mutagenesis and help in the spread of mobile genetic elements and pathogenicity islands (Úbeda et al., 2005), as discussed in subsequent sections.

Intracellular pH is regulated in *E. coli* cells by redox and proton pumps. However, a disturbance in pH regulation can lead to SOS induction (Padan et al., 1976; Padan and Schuldiner, 1986; Simmons et al., 2008). A mechanism for pH-induced expression of the SOS response is related to pH altering the structure and function of LexA (Dri and Moreau, 1994; van der Veen *et al.*, 2010). According to Sousa et al. (2006), in the low pH of 4.0, LexA has the tendency to self-aggregate, preventing its binding to the SOS box. Further in this condition, LexA has increased affinity for non-specific DNA, meaning that SOS box is also derepressed by titrating LexA to other DNA sequences in the genome. However, operons regulated by LexA are not transcriptionally active until mild condition (pH 5.0 - 6.0) is achieved, where cell metabolism is restored and LexA operators are still predominantly free of repression. The hypothesized mechanism would explain how the SOS response can be activated in a RecA independent manner to increase bacterial survival rate after an episode of stressful low pH condition.

High pressure also leads to DNA breaks and SOS response induction. In *E. coli* a mechanism for high-pressure-mediated DNA break has been linked to the expression of endogenous endonucleases that promote DSB after a high-pressure stress, which consequently triggers the SOS response (Aertsen et al., 2004). During food preservation processes bacterial pathogens are often exposed to high pressure to inactivate them, and SOS induction may contribute to their survivability (Alpas et al., 2000; Aertsen and Michiels, 2005; Simmons et al., 2008). 

## Diversity of the SOS response among bacterial species

*Escherichia coli* has served as the premier model from which almost all the fundamental aspects of SOS regulation and physiology have been derived. Nevertheless, it is now clear that the SOS response displays considerable variability among phylogenetically different bacteria. This variability is observed in two key aspects: the SOS box sequence (Table 1) and the set of genes repressed by LexA.

**Table 1 -:** Sequence of the SOS operator (SOS box) in different bacterial species.

Bacteria species	SOS box	Reference
*Bacillus subtilis*	CGAACN_4_GTTCG	Au et al., 2005
*Caulobacter crescentus*	GTTCN_7_GTTC	da Rocha et al., 2008
*Escherichia coli*	TACTG(TA)_5_CAGTA	Lewis et al., 1994
*Pseudomonas aeruginosa*	CTGN_2_TN7CAG	Cirz et al., 2006
*Staphylococcus aureus*	CGAACN_4_GTTCG	Cirz et al., 2007

Genes under LexA repression show variation between different bacterial species, however many cellular functions are commonly upregulated, for example genes encoding polymerases responsible for carrying out translesion synthesis, DNA repair proteins, cell division inhibitors, among others (Courcelle et al., 2001; da Rocha et al., 2008; Cirz et al., 2006, 2007).

The difference between the sequence recognized by LexA and the set of genes under its control seems to have an important role among species and how they respond to DNA damage (Erill et al., 2007). It can even be noted that it has already been described in some species, such as *Pseudomonas putida* and *Xanthomonas axonopodis* for example, the existence of two *lexA* regulons with independent LexA proteins and binding sequences (Yang et al., 2002; Abella et al., 2007). Furthermore, some bacteria also show SOS-independent DNA damage responses (e. g. Modell et al., 2014; Müller et al., 2018; Blanchard and Groot, 2021). 

The differences in the SOS boxes makes the regulator of one species unable to exert its function in other species (Lovett Jr et al., 1994), demonstrating their evolutionary importance, and being a possible factor that led to the formation of branches in the bacterial evolutionary tree (Mazón et al., 2004), since the LexA-binding sequence is monophyletic for phyla and classes (Erill et al., 2003). *P. aeruginosa* have consensus SOS box almost identical to the *E. coli* one and both are notably unrelated to the ones present in *Staphylococcus aureus* and *Bacillus subtillis*. These latter two species share similar SOS box consensus, with the LexA homolog being called DinR the regulator in *B. subtillis* (Table 1) (Winterling et al., 1997). On the other hand, the model organism *Caulobacter crescentus* has an SOS box composed of a direct repeat, which is found in other phylogenetically related bacteria (da Rocha et al., 2008). 

In *B. subtillis subtilis* only seven genes that are among the 33 genes under the control of LexA can be found in *E. coli* regulon composition. Under DNA damage, *P. aeruginosa* seems to upregulate the *recX* and *recN* whose gene products are recombination repair proteins, while *B. subtillis* upregulates *uvrBA* and *ruvAB* operons and *E. coli* upregulates all the genes cited above (Courcelle et al., 2003; Au et al., 2005). *C. crescentus* also shows upregulation in the expression of *recN*, *uvrA* and *ruvCAB* operon (da Rocha et al., 2008). On the other hand, *S. aureus* seems to downregulate the *recN* and *ruvBA* repair systems and upregulate *uvrBA* operon under damage induced by ciprofloxacin (Cirz et al., 2007).

The variation in the DNA damage response is illustrated by comparing the well-studied organisms *E. coli* and *P. aeruginosa*. The characterization of the SOS response in *E. coli* showed the derepression of 43 genes, in contrast with the 15 LexA-controlled genes in *Pseudomonas aeruginosa* (Courcelle et al., 2001; Cirz et al., 2006). Nevertheless, the response to DNA damage is more complex in *P. aeruginosa* because other regulons controlled by LexA-like repressors, with auto-cleavage promoted by activated RecA, are also induced alongside the canonical SOS response (Courcelle *et al.*, 2001; Cirz *et al.*, 2006). Such repressors are the PrtR protein - responsible for the repression of *prtN*, activator of the pyocin production (Matsui et al., 1993) and also required for expression of the type III secretion system (T3SS) through its repressive role on PtrB (Sun et al., 2014) - and the AlpR protein, which represses indirectly a self-lysis pathway promoted by the *alpBCDE* cluster (McFarland et al., 2015, Peña et al., 2021).

Two of the key aspects of the SOS response, cell division inhibition and translesion synthesis, show interesting variation in their main players when different bacteria are compared. Translesion synthesis and the consequent mutagenesis are mediated by error-prone DNA polymerases, mainly Pol V in *E. coli* (Goodman and Woodgate, 2013). Nevertheless, different bacteria use different SOS-regulated TLS pathways, as first evidenced by the characterization of DnaE2 and accessory proteins (Boshoff et al., 2003; Galhardo et al., 2005), as discussed in the next section. 

To avoid DNA replication and segregation problems, the SOS response activates inhibitors of cell division. The cell division in *E. coli* under DNA damage stops when the product of *sulA* gene interacts with FtsZ and inhibits its GTPase activity (Trusca et al., 1998). FtsZ is a GTP-binding protein abundant during the early stage of cell division, responsible for polymerizing a ring structure in the middle of the bacterial cell where the future separation of cells occurs in normal conditions (De Boer et al., 1992). It has already been shown that the SulA protein also interacts with FtsZ in *P. aeruginosa*, however the ability to inhibit cell division per se has not yet been confirmed (Cordell et al., 2003). In SOS-inducing conditions, *C. crescentus* upregulates the *imuA* gene that shows weak, but enough homology to be confounded with *sulA* in a few bacterial genomic annotations, like in *Pseudomonas putida*, for example (Erill et al., 2006; McHenry, 2018). Yet, it is known that in *C. crescentus*, the filamentation caused by DNA-damage occurs through the inhibition of the final step of cell division by the interaction of a small inner membrane protein, product of *sidA* gene, with FtsW, one of the proteins responsible for cell constriction (Modell et al., 2011). In *B. subtillis* the inhibition of cell division occurs through YneA, also a membrane protein, that when expressed upon SOS-inducing conditions, promotes cell elongation (Kawai et al., 2003). However, the FtsZ ring is still polymerized, so YneA acts via protein-protein interaction with proteins, other than FtsZ, that could be part of the divisome, therefore differing in activity from SulA (Mo and Burkholder, 2010). *S. aureus* displays a similar mechanism where the SosA membrane protein inhibits the division septum formation causing filamentation probably through interaction with proteins responsible for a later stage of the division like in *B. subtillis* (Bojer et al., 2019).

Characterization of the SOS regulon of *C. crescentus* (da Rocha et al., 2008; Modell et al., 2011) exemplifies how the study of the SOS response in different bacterial species may reveal novel aspects of prokaryotic DNA repair and cellular defense mechanisms. Two SOS-regulated genes (*mmcA* and *mmcB*) were identified as agents protecting cells from Mitomycin C, a cross-linking agent. MmcA is probably a detoxifying enzyme, while MmcB is an endonuclease from the PD-(D/E)XK family (Lopes-Kulishev et al., 2015), also mediating resistance to cisplatin (Price et al., 2018). MmcB has been hypothesized to participate in a repair pathway also involving translesion synthesis polymerases to allow removal of interstrand crosslinks (Lopes-Kulishev *et al.*, 2015). Another pair of SOS-regulated genes encode a toxin-antitoxin system (HigAB). The RNAse activity of the toxin HigB targets key mRNAs, therefore acting as a growth regulator after DNA damage (Kirkpatrick et al., 2016).

Besides the difference in LexA binding sites and set of regulated genes, the regulator itself can also vary between species. In the *Streptococcacea* family, SOS response is regulated by the HdiR repressor, a peptidase of the S24-family such as LexA, which similarly to LexA has the ability to self-cleave in the presence of ssDNA-RecA and release the transcription of an SOS regulon composed basically of error-prone polymerases (Savijoki et al., 2003). The same occurs in the *Moraxellaceae* family but the regulator is the UmuDab protein (Hare et al., 2014). In the phylum of Bacteroidetes, the SOS response is regulated by a new peptidase from the S24-family of phage-like repressors which, when derepressed, activates the expression of standard SOS genes (Sánchez-Osuna et al., 2021). The evolution of these peptidases with independent DNA-binding domains once again shows how heterogeneous this response can be.

## Translesion synthesis, mutagenesis and bacterial evolution

One of the most intensely studied aspects of the SOS response is its influence on mutagenesis. Early studies on mutagenesis induced by ultraviolet radiation have led to the recognition that mutations are not always the result of passive replication errors caused by mutagens - on the contrary, these mutations are the result of active processing of DNA damage by the cellular machinery (reviewed by Friedberg et al., 2006). This fascinating concept has emerged from studies by Jean Weigle, in which UV irradiated λ bacteriophage was shown to have improved survival if the host cells had been pre-irradiated as well. In the same way, mutagenesis resulting from such irradiation of phages with UV light was only observed if the host cells had been pre-irradiated (Weigle, 1953). These phenomena were named respectively “Weigle reactivation” and “Weigle mutagenesis”. These observations led to the correct conclusion that mutagenesis requires an active processing of the damaged DNA by cells, which is mediated by an inducible cellular component. 

This is a consequence of translesion DNA synthesis (TLS) polymerases, one of the pathways repressed by LexA and regulated by the SOS response. All living organisms are dependent on DNA polymerases for efficiently replicating their genetic material, however DNA damage causes blockage of the replisome and induction of the SOS response, a situation that can be circumvented by TLS-polymerases. These polymerases lack proofreading exonuclease activity and are error-prone, leading to incorporation of incorrect nucleotides (Goodman and Woodgate, 2013). However, their flexible active sites and additional little finger domain allow them to achieve TLS, using damaged DNA as templates and continuing replication (Boudsocq et al., 2004; Friedberg et al., 2006). Even though this process is essential for bacterial survival in adverse conditions, it could be detrimental due to the generation of deleterious mutations, making it crucial for bacteria to tightly regulate induction of TLS. On the other hand, it also may lead to bacterial evolution and diversity in virtue of mutagenesis (Galhardo et al., 2007; Goodman and Woodgate, 2013; Zhang, 2020). In fact, recent findings suggest that *E. coli* cells may use TLS as the first choice to deal with replication blockage, rather than error-free damage avoidance pathways, favoring the generation of genetic variability (Naiman et al., 2014).

In *E. coli*, three DNA polymerases are regulated by LexA: Pol II (*polB*), Pol IV (*dinB*) and Pol V (*umuDC*) (Courcelle et al., 2001), all of which are involved in mutagenesis to some extent (Napolitano et al., 2000). The induction of *polB* and *dinB* occurs early in the SOS response, related to the weak binding of LexA (Fernández de Henestrosa et al., 2000). These are responsible for TLS in specific DNA damages, in contrast to *umuDC*, considered as much more error-prone and able to bypass a more diverse set of DNA lesions, used as last resource and being strongly regulated (Sommer et al., 1993; Fernández de Henestrosa *et al.*, 2000).

Pol II (*polB*) is a B-family polymerase that had its TLS function, bypass abasic lesions (Bonner et al., 1988), unveiled years after its first characterization by Knippers (1970), with low involvement in mutagenesis. UmuC and DinB are members of the Y-family of DNA polymerases, which includes many bacterial, archaeal and eukaryotic enzymes (Ohmori et al., 2001; reviewed by Jarosz et al., 2007).

Although a physiological role for DinB in DNA damage tolerance was harder to identify on the basis of phenotypes of a *dinB* mutant strain, it has been implicated in tolerance to some types of DNA damage, especially adducts in position N^2^ of guanines and alkylative lesions (Kim et al., 2001, Jarosz et al., 2006; Bjedov et al., 2007). DinB has an error rate between 10^-3^ and 10^-5^ *in vitro* (Tang et al., 2000; Jarosz *et al.*, 2007) when using a non-damaged DNA as a substrate. Overexpression of *dinB* is heavily mutagenic to *E. coli*, introducing mainly -1 frameshifts at G:C runs (Kim *et al.*, 1997), the same being observed in *in vitro* gap filling assays using the *lacZ* gene as a target (Kobayashi et al., 2002). Mutagenesis caused by overexpression of DinB occurs preferentially in the lagging strand (Kuban et al., 2005), a smaller but significant number of base substitutions are also observed. 

*E. coli* DinB promotes TLS across adducts in the N^2^ position of guanine with high efficiency and accuracy (Jarosz et al., 2007). Genetic data also indicate that DinB takes place in error-free TLS in sites of endogenous alkylation damage that accumulates in repair-deficient strains (Bjedov et al., 2007). Lastly, *dinB* plays a major role in the process of stress-induced mutagenesis in non-growing cells (Mckenzie et al., 2001; Galhardo et al., 2009). 

DinB is expressed as part of an SOS-regulated operon, which also contains the *yafN-yafO* toxin-antitoxin system and *yafP* (Singletary et al., 2009). The *yafP* gene encodes a putative acetyl-transferase probably involved in the metabolic transformation of genotoxic compounds (Gutierrez et al., 2011). Interestingly, *umuDC* is tightly repressed in SOS-uninduced cells, whereas *dinB* has a significant basal level of expression. In fact, about 250 molecules of DinB are present in cells, in contrast to only about 10-20 molecules of the holoenzyme of DNA Pol III, the enzyme responsible for normal replication (Fijalkowska et al., 2012). Upon SOS induction, the number of DinB molecules rises 10-fold to about 2500 molecules per cell (Kim et al., 2001). DinB expression and activity are subject to several levels of control. The *dinB* gene is also induced independently of the SOS response both as part of the stationary phase regulon controlled by the alternative sigma factor RpoS (Layton and Foster, 2003) and after exposure to beta-lactam antibiotics (Pérez-Capilla et al., 2005). Activity of this polymerase is modulated by a plethora of interactions, including UmuD, polyphosphate kinase (ppk), Rep helicase, RecA and the transcription elongation factor NusA (Stumpf and Foster, 2005; Godoy et al., 2007; Cohen et al., 2009; Sladewski et al., 2011). 

Pol V (*umuDC*) is highly mutagenic, being considered the most important TLS-polymerase according to its capacity to bypass diverse forms of DNA lesions (Goodman and Woodgate, 2013). In accordance, this is the most studied TLS-polymerase, with orthologs identified in diverse prokaryotes and mobile genetic elements (Vaisman et al., 2012), such as the homologs *mucAB* described in plasmids (Perry and Walker, 1982) and *rumAB* in integrative and conjugative elements (ICEs) (Kulaeva et al., 1995). The function of UmuDC was first observed in the 70s, by Miroslav Radman and Evelyn Witkin (Radman, 1974; Sikand et al., 2021), although at that time the specific polymerase responsible for the mutagenic activity in the SOS response had not been elucidated (Sikand *et al.*, 2021). Genetic identification of *umuDC* genes was first reported in a search for *E. coli* strains lacking UV-inducible mutagenesis (Kato and Shinoura, 1977). In the 80s *umuC* and *umuD* genes were revealed as an operon regulated by LexA and RecA (Bagg et al., 1981; Elledge and Walker, 1983; Shinagawa et al., 1983), but only in the late 90s purification and study of the mutagenic activity of UmuDC were achieved (Bruck et al., 1996; Tang et al., 1998, 1999; Reuven et al., 1999).

The complex modulation of DNA Pol V also involves the RecA protein. RecA is necessary both for the induction of the SOS response and for UmuD cleavage, in a process similar to what occurs to LexA with the involvement of RecA*. RecA* induces self-cleavage of UmuD in UmuD’, forming the complex with UmuC - UmuD’_2_C (Pol V) (Jiang et al., 2009). Additionally, early genetic studies have shown that RecA performs a third role in *umuDC*-dependent SOS mutagenesis (Blanco et al., 1982; Nohmi et al., 1988; Dutreix et al., 1989; Sweasy et al., 1990). *In vitro* experiments have shown that RecA bound to ssDNA is necessary for mutagenesis, with latest models suggesting that the “mutasome” complex operating in TLS is a molecular assembly of UmuD’_2_C-RecA-ATP (reviewed by Fujii and Fuchs 2020; Jaszczur et al., 2016; Sikand et al., 2021). RecFOR proteins also have a role in the formation of the RecA filament necessary for UmuD’_2_C TLS (Fujii *et al.*, 2006). The mutagenic activity of Pol V is not only capable of incorporating incorrect nucleotides into DNA lesions, but also upstream and downstream of it (Maor-Shoshani et al., 2000; Isogawa et al., 2018; Fujii and Fuchs, 2020).

Bacteria that do not possess Pol V, approximately two thirds of the bacteria with known genomes (Sheng et al., 2021), may possess an SOS cassette consisting of *imuABC* (*imuAB* and *dnaE2*), responsible for TLS and mutagenic activity in stressing conditions, mainly distributed among Proteobacteria (Galhardo et al., 2005; Erill et al., 2006; McHenry, 2011). However, it is important to emphasize that genetic composition and configuration of this cassette is variable among bacterial species, some of them lacking *imuA* or with different genes supporting DnaE2 activity (Erill *et al.*, 2006; Timinskas and Venclovas, 2019; Blanchard and Groot, 2021). 

The relation of *imuC* (*dnaE2*) with mutagenic activity was first established in studies with *Mycobacterium tuberculosis* (Boshoff et al., 2003). Additionally, it was shown that *dnaE2* is co-transcribed with *imuA* and *imuB* in *C. crescentus* and a reduced damage-induced mutagenesis activity was observed when any of these three genes were deleted (Galhardo et al., 2005). Later, a role for *imuABC*-like cassettes in damage-inducible mutagenesis and DNA damage tolerance was confirmed in other bacterial species (Koorits et al., 2007; Zeng et al., 2011; Blanchard and Groot, 2021; Sheng et al., 2021). In contrast to its role in TLS, involvement of ImuC in spontaneous mutagenesis in *C. crescentus* is minor (Valencia et al., 2020), and not enhanced by a constitutively transcribed *imuABC* operon (Alves et al., 2017). More recently it has been shown that non-dividing *C. crescentus* cells employ ImuC in DNA synthesis during gap filling of nucleotide excision repair intermediates (Joseph et al., 2021).

ImuA is a protein distantly related to SulA and RecA, ImuB is a catalytically dead Y-family polymerase, whereas ImuC (DnaE2) is a paralog of the Pol III´s alpha subunit without proofreading exonuclease activity, consequently error-prone and SOS-mutagenic (Galhardo et al., 2005; Warner et al., 2010; Timinskas et al., 2014). In *M. tuberculosis*, ImuC mutagenesis is also dependent on ImuA and ImuB supporting activity, ImuB being responsible for making the connection of ImuC with the β-clamp in the replication fork, making possible for ImuC to continue its function (Warner *et al.*, 2010). Unlike SOS mutagenesis in *E. coli*, ImuABC activity in *C. crescentus* is independent of RecA, which leads to the hypothesis that ImuA may perform a similar role as the former in TLS (Alves et al., 2017). Recent results obtained in *Myxococcus xanthus* revealed that ImuA does not bind DNA, but interferes with RecA activity, which may indicate that this protein has a role in inhibiting competing pathways such as homologous recombination (Sheng et al., 2021).

The mutagenic activity of translesion DNA polymerases may be described as targeted (damaged DNA) or untargeted (undamaged and distant DNA sites), these events are constantly checked by DNA mismatch repair (MMR) systems, as a form of preventing misincorporations and mutations after the replication (Lewis et al., 2021). However, one of the most intriguing consequences of TLS-polymerases action is the phenomenon of antibiotic-induced mutagenesis. Antimicrobial agents of different types of action, and of regular clinical usage, are involved in the induction of the SOS response by ROS generation (Kohanski et al., 2007; Dwyer et al., 2014; Memar et al., 2018; Crane et al., 2021), consequently triggering the hypermutation phenotype and bacterial evolution that TLS polymerases may potentiate, including mutations that cause acquisition of adaptive mechanisms and resistance to antibiotics (Goodman, 2016; Memar *et al.*, 2020). The contribution of ROS to bacterial killing by antibiotics is still under debate (Liu and Imlay, 2013; Keren et al., 2013), but it has become increasingly clear that antibiotics, at least in part through ROS generation, induce an SOS-dependent increase in mutagenesis (Pribis et al., 2019; Rodríguez-Rosado et al., 2019). 

## Targeting the SOS DNA repair system as a countermeasure to antibiotic resistance

The rise of antibiotic resistant bacteria poses an unprecedented concern since the discovery of penicillin (Sengupta et al., 2013). The underlying mechanism for the increasing threat is related to the large amount and misuse of antibiotics in agricultural/livestock production and therapy, where a range of sub-lethal antibiotic concentrations are released in the environment (Mann et al., 2021). Beta-lactams, quinolones and aminoglycosides are known to ultimately produce ROS in bacteria, which can directly damage proteins, DNA and cell membrane (Kohanski et al., 2007). However, while sub-therapeutic concentrations of antibiotics are not sufficient to kill bacteria, they still stimulate the SOS response by DNA damage (Kohanski *et al.*, 2010; Thi et al., 2011). SOS increases the number of mutational events by upregulating error-prone TLS polymerases (Boshoff et al., 2003) and stimulates horizontal gene transfer (Beaber et al., 2004; Crane et al., 2018), biofilm formation (Gotoh et al., 2010) and the appearance of small colony variants, all of which have the potential to increase tolerance against antibiotics (Memar et al., 2020; Podlesek and Bertok, 2020).

It has been shown that combining antibiotics and suppression of the SOS response decreases the formation of resistant strains (e. g. Cirz et al., 2005; Thi et al., 2011; Recacha et al., 2017, Valencia et al., 2017). The most studied approaches to block the SOS response are prevention of either the activation of RecA protein or the autocatalysis of LexA cleavage. There are different alternatives to interfere with RecA activity, for example, disturbing proper filament RecA-ssDNA formation (Lee et al., 2005; Petrova et al., 2009; Nautiyal et al., 2014), or affecting the RecA ATP binding/ATPase activity that is necessary for its activation (Wigle and Singleton, 2007; Bellio et al., 2017; Ojha and Patil, 2019). Both strategies affect RecA-dependent LexA proteolysis, thus blocking the SOS response.

However, RecA has homology to a human recombinase Rad51 (Kawabata et al., 2005). This raises a concern on the usage of these compounds in combination with antibiotics. A better alternative would be to target LexA, as there are no corresponding orthologs in the human genome. A study found that phenylboronic derivatives could interfere with LexA self-cleavage by forming an acyl-enzyme intermediate with the catalytic Ser-119 (Bellio et al., 2020). Nevertheless, research on SOS inhibition directly affecting LexA is still very scarce.

Although no drug targeting the SOS machinery has been approved yet, there is no doubt that suppressing evolutionary mechanisms responsible to increase tolerance against bactericidal agents is a very promising approach to extend the shelf life of antibiotics in use today.

## Relationship between SOS response and mobile genetic elements

**Figure 2 -:**
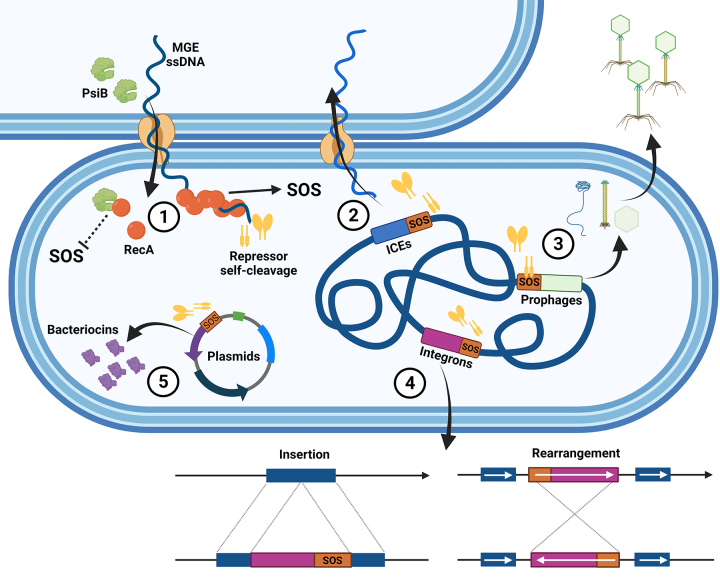
Schematic representation of the involvement of SOS response with mobile genetic elements (MGE). (1) Entry of mobile elements ssDNA in the host cell induces the SOS response by the formation of RecA* filaments, however some MGE encode proteins (such as PsiB) that are able to bind free RecA, avoiding all functions of RecA including the initiation of the SOS response. (2) The SOS response regulates the transfer of integrative and conjugative elements (ICEs) from the SXT/R391 family by interacting with SetR, repressor that self-cleaves after RecA* stimulus. (3) Bacteriophages may go from lysogeny to lytic cycle after induction of the SOS response, some phages show an SOS box sequence on promoter regions, others may encode represors, like the lambda bacteriophage CI repressor that self-cleaves after RecA* stimulus. (4) Chromosomal and mobile integrons show SOS box sequences in promoter regions, with transfer and rearrangement in the chromosome after the SOS response induction. (5) The SOS response regulates expression of bacteriocins, enforcing the maintenance of plasmids in the host cell.

When lysogenized bacteria undergo DNA damage, bacteriophages switch to the lytic cycle, presumably to escape from an endangered host and disperse in the environment (Little, 2005). This early observation in phage biology underlies a phenomenon shared by other mobile genetic elements (MGEs), such as integrons (Guerin et al., 2009), chromosome cassettes (Liu et al., 2017), pathogenicity islands (Chittò et al., 2020) and integrative and conjugative elements (ICEs) (Beaber et al., 2004; Auchtung et al., 2005). Figure 2 depicts the relationship of the SOS response with MGEs.

The SOS regulators RecA and LexA are both involved in the regulation of MGEs transfer (Fornelos et al., 2016). Several chromosomal and mobile integrons show SOS box sequences in promoter regions (Guerin et al., 2009), as well as ICEs that may possess repressors regulated by RecA (Beaber et al., 2004), consequently showing that induction of the SOS response also regulates MGEs transfer and chromosomal rearrangement during conjugation (Baharoglu et al., 2010).

It was initially observed that DNA damage was able to cause changes in the life cycle of temperate phages, from lysogeny to the lytic cycle (Little, 2005). Multiple phages are SOS-induced and regulated by LexA, others use their own RecA-controlled repressors in a similar mechanism to the self-cleavage of LexA by RecA*. The most prominent example is the λ phage that is maintained integrated in the chromosome through the CI repressor and when there is DNA damage, RecA* induces autocleavage of CI and expression of λ phage genes (Hochschild and Lewis, 2009; Fornelos et al., 2016).

ICEs from the SXT/R391 family encode the SetR repressor, from the same family of the λ-CI repressor, showing self-cleavage activity regulated by RecA in the SOS response (Beaber et al., 2004; González et al., 2019). SetR is responsible for repressing *setCD*, two genes involved in the transfer of ICEs (Beaber *et al.*, 2002, 2004), and, along with LexA and CroS, also regulates the mutagenic activity of the *umuDC* homologs *rumAB* encoded in the ICE (González *et al.*, 2019; McDonald et al., 2021). It is interesting to note that some species that often carry SXT/R391 elements such as *Proteus mirabilis*, are naturally devoid of chromosomal *umuDC* genes. This species is accordingly non-mutable by UV irradiation, but acquisition of SXT/R391 elements provides TLS and mutagenesis capacity to this bacterium. Furthermore, *rumAB* genes improve conjugation of the ICE to new hosts (Sato et al., 2022), demonstrating an intricate relationship of these MGEs with the SOS response.

LexA is not only able to mediate control over MGEs horizontal transfer but also over the expression of virulence factors and bacteriocins carried by MGEs (Fornelos et al., 2016). For example, clusters present in plasmids are responsible for the expression of the toxic colicin protein, which is capable of killing competing bacteria and enforcing the maintenance of the plasmids in the host through the expression of immunity proteins (Cascales et al., 2007; Budič *et al.*, 2011; Fornelos *et al.*, 2016). An interesting observation was also made by Kamruzzaman and Iredell (2019) that conjugative plasmids (mainly IncI and IncF) benefited from a toxin-antitoxin system (*parDE* ^*I*^ ) that is induced by stress and also elicits the SOS response, that provides antibiotic tolerance and allows the plasmid to successfully stabilize in the bacterial cell.

Diverse MGEs influence the host’s SOS response. The acquisition of new MGE is a distress event that induces the SOS response, caused by the filamentation of RecA in the ssDNA - intermediate form of transfer of MGEs - mainly in DNA with low homology to the chromosome (Baharoglu et al., 2010; Al Mamun et al., 2021). There is also development of systems to repress the SOS response so that the MGE can successfully integrate or perpetuate itself in the new host (Memar et al., 2020; Al Mamun *et al.*, 2021). It has been shown that SOS inhibiting proteins, such as PsiB (Bagdasarian et al., 1992; Petrova et al., 2009) and SSB, are translocated through the secretion system (T4SS) together with the MGEs ssDNA, which facilitates the maintenance of these elements (Al Mamun *et al.*, 2021). The regulation of frequency of transmission is also important for the survival of the MGE in observation that excess of horizontal transfer causes impact in the host cell (Touchon et al., 2014). 

Overall, the SOS response protects cells from DNA-damaging environmental stressors and is a main player in the acquisition of antimicrobial resistance through mutagenic activity and induction of horizontal transfer of MGEs carrying these traits.
